# Retraction Note: A biomimetic hybrid nanoplatform for encapsulation and precisely controlled delivery of theranostic agents

**DOI:** 10.1038/s41467-026-74778-3

**Published:** 2026-06-26

**Authors:** Hai Wang, Pranay Agarwal, Shuting Zhao, Jianhua Yu, Xiongbin Lu, Xiaoming He

**Affiliations:** 1 Department of Biomedical Engineering, The Ohio State University, Columbus, Ohio 43210 USA; 2 Comprehensive Cancer Center, The Ohio State University, Columbus, Ohio 43210 USA; 3 Davis Heart and Lung Research Institute, The Ohio State University, Columbus, Ohio 43210 USA; 4 Division of Hematology, The Ohio State University, Columbus, Ohio 43210 USA; 5 Department of Cancer Biology, University of Texas MD Anderson Cancer Center, Houston, Texas 77030 USA

Retraction to: *Nature Communications* 10.1038/ncomms10081, published online 01 December 2015

This paper is being retracted after a joint research misconduct investigation conducted by Indiana University, The Ohio State University, and the University of Maryland, College Park found that Figure 4a and Supplementary Fig. 22 contain irregularities. The Editor has therefore lost confidence in this paper.

Xiaoming He disagrees with this retraction, Hai Wang, Pranay Agarwal, Shuting Zhao, Jianhua Yu and Xiongbin Lu did not respond to correspondence from the Publisher about this retraction.

